# Intrinsic Néel Antiferromagnetic Multimeronic Spin Textures in Ultrathin Films

**DOI:** 10.1021/acs.jpclett.3c02419

**Published:** 2023-09-29

**Authors:** Amal Aldarawsheh, Moritz Sallermann, Muayad Abusaa, Samir Lounis

**Affiliations:** † Peter Grünberg Institute and Institute for Advanced Simulation, Forschungszentrum Jülich and JARA, D-52425 Jülich, Germany; ‡ Faculty of Physics, University of Duisburg-Essen and CENIDE, 47053 Duisburg, Germany; § RWTH Aachen University, 52056 Aachen, Germany; ∥ Science Institute and Faculty of Physical Sciences, University of Iceland, VR-III, 107 Reykjavík, Iceland; ⊥ Department of Physics, Arab American University, 240 Jenin, Palestine

## Abstract

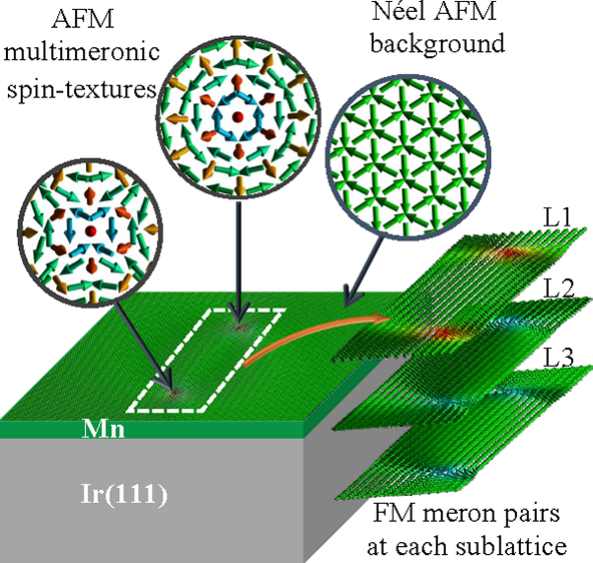

Topological antiferromagnetism is a vibrant and captivating research field, generating considerable enthusiasm with the aim of identifying topologically protected magnetic states of key importance in the hybrid realm of topology, magnetism, and spintronics. While topological antiferromagnetic (AFM) solitons bear various advantages with respect to their ferromagnetic cousins, their observation is scarce. Utilizing first-principles simulations, here we predict new chiral particles in the realm of AFM topological magnetism, exchange-frustrated multimeronic spin textures hosted by a Néel magnetic state, arising universally in single Mn layers directly grown on an Ir(111) surface or interfaced with Pd-based films. These nanoscale topological structures are intrinsic; i.e. they form in a single AFM material, can carry distinct topological charges, and can combine in various multimeronic sequences with enhanced stability against external magnetic fields. We envision the frustrated Néel AFM multimerons as exciting highly sought after AFM solitons having the potential to be utilized in novel spintronic devices hinging on nonsynthetic AFM quantum materials, further advancing the frontiers of nanotechnology and nanophysics.

Recent experimental breakthroughs promoted antiferromagnetic (AFM) materials into the realm of information technological applications^1−9^ and triggered state-of-the-art activities in the world of topological magnetism.^7,10−13^ The antiparallel spin sublattices present in AFM materials result in zero dipolar fields, making them insensitive to magnetic field perturbations and enhancing the stabilization of nanoscale topological structures.^1,4,9,10,14−16^ Moreover, AFM materials possess faster spin dynamics than ferromagnets by orders of magnitude,^17−20^ which is an appealing characteristic for THz magnetic memory and logic devices.

The race in identifying AFM nontrivial spin-swirling objects is strongly motivated by their particle-like nature, potentially realizing ideal magnetic bits, augmented with the low power consumption^4,7,12,14,21−34^ involved in their manipulation with the possibility of controlling their current-driven motion^9,14,16,22,23,25,26,28^ while avoiding the skyrmion Hall effect that plagues the ferromagnetic (FM) cousins.^35−39^

This led to the recent discovery of synthetic AFM skyrmions, which consist of two FM skyrmions that are realized in two distinct magnetic layers and antiferromagnetically coupled through a nonmagnetic spacer layer.^7,10−13^ Here, utilizing first-principles in conjunction with atomistic spin dynamics, we unveil multimeronic textures, a new type of topological AFM particles, which are nonsynthetic and emerge in magnetically frustrated thin films (see Figure 1). Therefore, and importantly, these findings are distinctly set apart from previously experimentally discovered or theoretically predicted AFM solitons, carrying the potential to revolutionize the fields of nanotechnology and nanoscience by offering unprecedented opportunities for designing and engineering cutting-edge magnetic nanomaterials with tailored properties.

**Figure 1:**
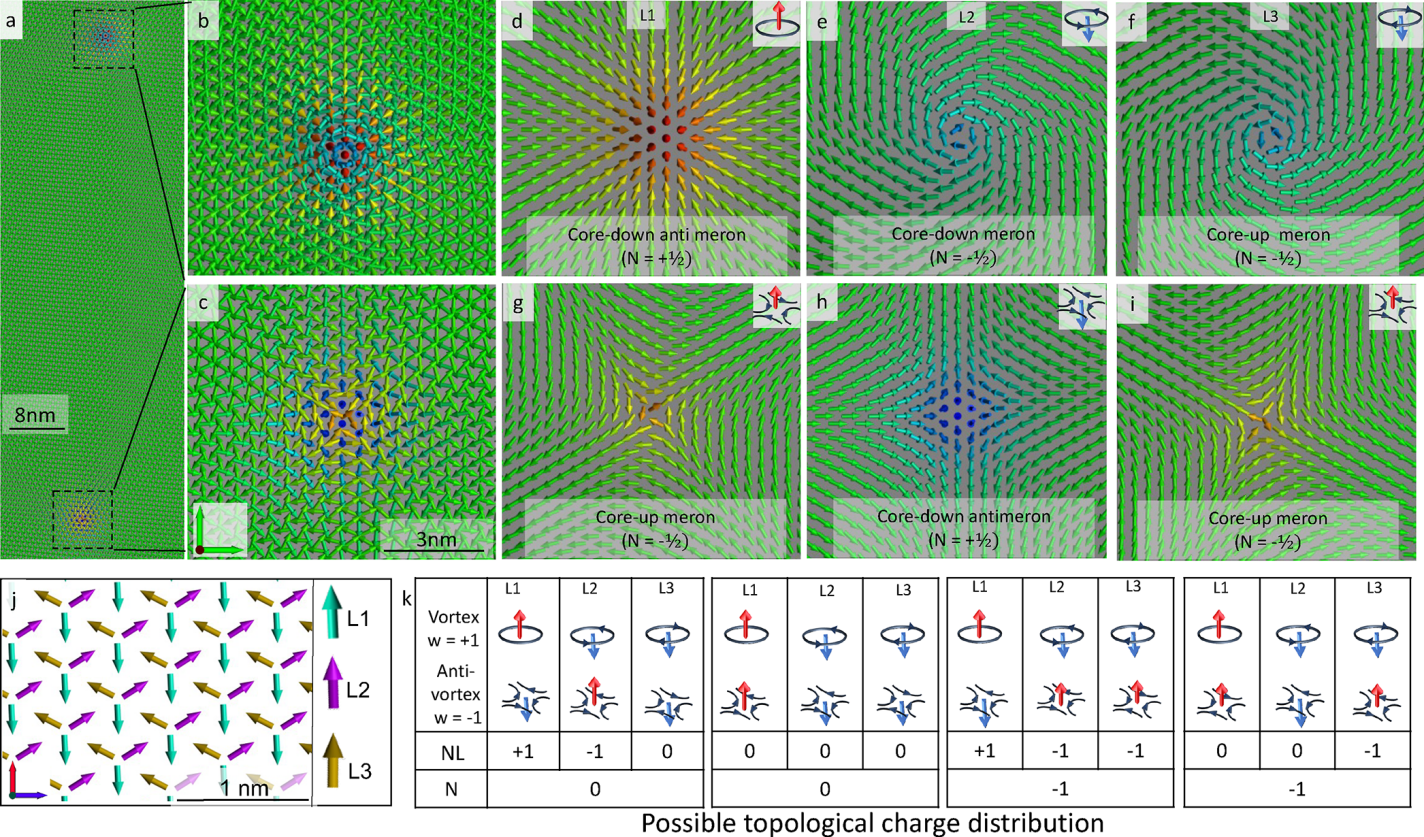
Frustrated Néel AFM meronic topology: (a) AFM hexameronic state composed of a vortex–antivortex pair that emerges in a frustrated triangular Mn layer on e.g. the Ir(111) surface with zoom into the vortex (b) and antivortex (c) components. The frustrated AFM meronic texture is decomposed into three FM vortix–antivortex pairs residing at sublattices L1 (d, g), L2 (e, h), and L3 (f, i). (j) Illustration of the Néel AFM ground state with colors indicating the decomposition into three sublattices L1, L2, and L3. (k) Schematic representation of the set of sublattices for the possible topological magnetic structures, where *w* stands for the winding number and *N* stands for the topological number (*N* = *wp*/2 with *p* = +1 for up and *p* = −1 for down polarity of the core).

Regular FM merons are in-plane magnetized textures with magnetization that curls around a stable core pointing out-of-plane and are topologically equivalent to one-half of a skyrmion.^40−49^ The meronic topological charge  equals , where **n** is the direction vector of magnetization. They have been observed experimentally in thin films^45,50^ and in bulk as cross sections of vortex–antivortex three-dimensional rings.^51^ Antiferromagnetically coupled merons emerge synthetically in confined geometries^42,44^ or nucleate across domain walls.^52,53^ They were identified in hybrid complexes involving various magnetic objects in intrinsic bulk (thick films) phases,^8,31,54^ following a large body of phenomenology-based simulations.^30,32−34,55^ However, a pristine ultrathin film material that hosts AFM merons remains elusive.

Our multimeronic textures are distinct from current predictions because they form in a realistic material a rich set of combinations materializing in a frustrated in-plane Néel ground state, shown in Figure 1j, which can be decomposed into three FM sublattices, with an opening angle of 120° between their respective magnetic moments. We predict a single Mn layer as a universal hosting material once interfaced in different fashions with the Ir(111) surface with and without Pd and Fe monolayers, PdFe bilayer, or Pd_2_Fe trilayer (see Figure 2a–d). The different substrates form a typical family of substrates typically known to host FM^56−66^ and AFM skyrmions.^9,16^ The in-plane AFM Néel state is the ground state for the Mn layer in all magnetic systems, formed as a result of magnetic frustration caused by strong AFM exchange coupling among the first nearest neighbors, as illustrated in Figure S1. The in-plane orientation is dictated by the *z* component of the antisymmetric exchange interactions (Dzyaloshinskii–Moriya interactions, DMI) and is further reinforced by the in-plane magnetic anisotropy energy (MAE) *K*. In the evolving realm of topological antiferromagnetism, our research unveils novel nanoscale topological AFM solitons that emerge in a set of different magnetic states. As the nanotechnology landscape progresses, our findings hold the potential to revolutionize information technology by propelling the use of AFM topological concepts, thereby influencing both a fundamental understanding and revolutionary nanotech applications.

**Figure 2:**
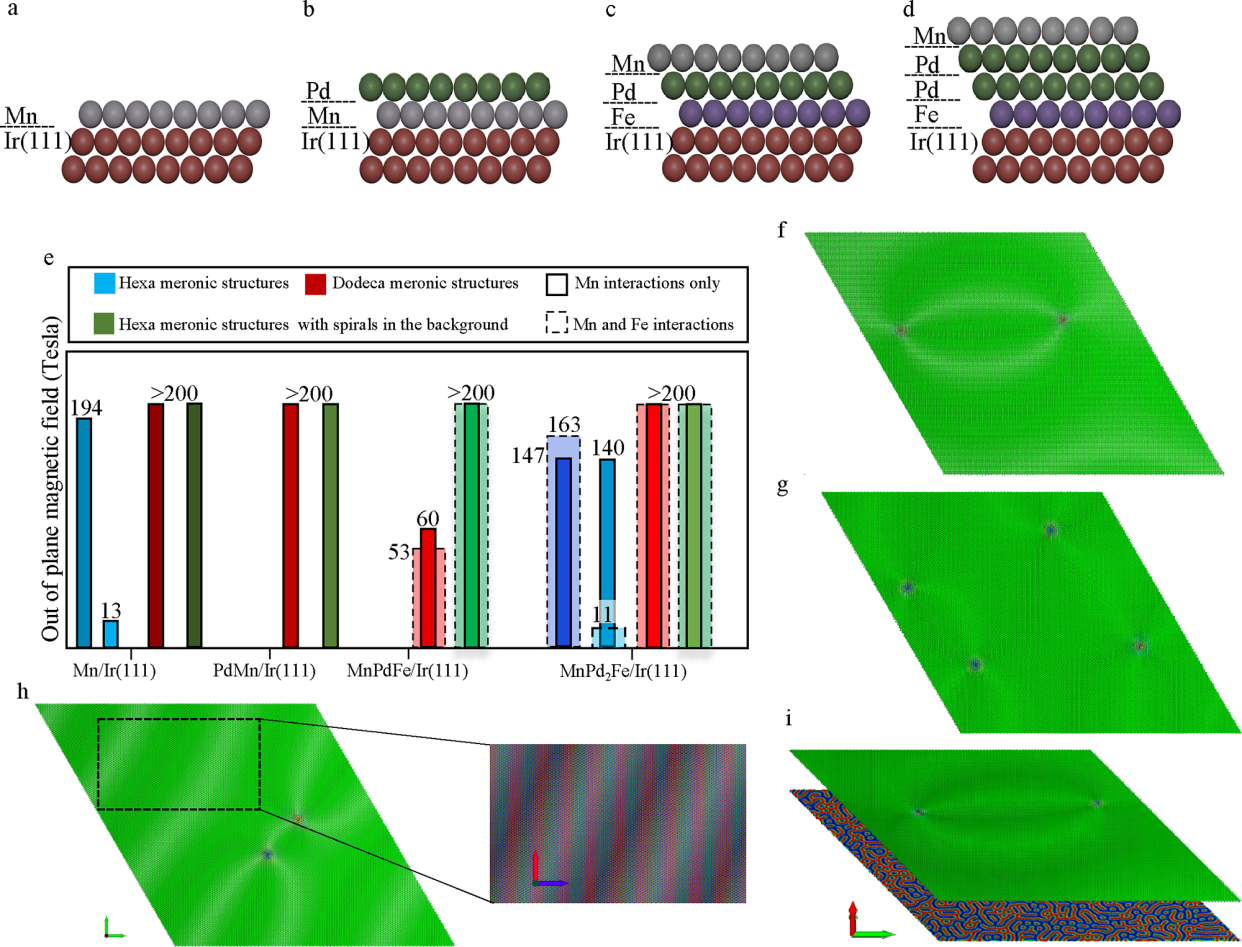
Impact of external magnetic field: (a–d) Schematic representation of the magnetic layered systems of study. (e) The critical out-of-plane magnetic field annihilating our multimeronic textures. Blue and red bars refer to respectively hexa- and dodecamerons emerging in AFM Néel order, while green bars correspond to hexamerons hosted by initially spiraling Néel states. Bars with solid versus dashed lines distinguish the cases without and with Mn–Fe interactions. (f–h) Snapshots for the blue, red, and green magnetic states, respectively; the inset shows the spiral at the background of (h). (i) Snapshot showing the blue dashed bar representing the Mn layer (upper layer) interfaced with the Fe layer (lower layer) in MnPd_2_Fe.

## Topological Magnetic States in Frustrated Mn Layer

The Ir(111) substrate forms a triangular lattice, on which we deposit layers of Mn, PdMn, MnPdFe, and MnPd_2_Fe and perform atomistic spin dynamics,^67^ minimizing the Heisenberg Hamiltonian (eq 1 in the Methods section) equipped with the magnetic interactions derived from first-principles (see the Methods section). We identify a plethora of AFM Néel meronic magnetic states forming metastable states in the Mn layer, as depicted in Figure 1a, Figure 2g,h, and Figure S2.

The Néel ordering of the spins is the ground state of the Mn layer in all of the aforementioned magnetic systems. The associated critical temperatures range from 130 K for PdMn bilayers to about 600 K or more for the rest of the explored Mn-based films. The spins forming the AFM Néel order are segmented into three sublattices L1, L2, and L3, each hosting FM spin alignment (Figure 1j). At each sublattice, the FM meronic pair can be stabilized, so in total, in the case of the single AFM Néel meronic pair (Figure 1a), we have six FM merons (antimerons), as shown in Figure 1d–i, which we refer to as a hexameronic state. By zooming in on the two spin-swirling extremities of the hexameron (Figure 1b,c) and their respective sublattice decomposition (Figure 1d–i), we identify a vortex (Figure 1d) and an antivortex (Figure 1h) whose cores reside on an Mn lattice site, around which the spins of the remaining meronic textures precess, as dictated by the magnetic frustration induced by the underlying AFM magnetic interactions.

Each of the FM building blocks of our AFM explored solitons holds a topological charge (*N*) defined as *N* = (*wp*/2),^40^ where *w* = +1 (−1) for the vortex (antivortex) is the winding number describing the rotational direction of the in-plane magnetization and *p* is the polarity which defines the out-of-plane magnetization of the center being +1 (−1) when pointing up (down).^68^ Because the merons and antimerons carry a topological charge of −1/2 and +1/2, respectively,^31,45,50^ the sublattice charge *N*_L_ is either −1 (+1) for a meron–meron (antimeron–antimeron) pair, as the case of L3 (Figure 1f,i), or 0 for a hybrid (see L1 and L2 in Figure 1d,e,g,h) meron–antimeron pair. By summing up the total charge *N*_t_ for a hexameron, one can end up with three possible values: −1, 0, and +1 (see Figure 1k), which interestingly are energetically degenerate in the absence of an external magnetic field.

Besides the hexameronic frustrated AFM Néel state, we identified a rich set of other meronic textures, such as the dodecameron, hosting 12 merons, shown in Figure 2g. Further examples of complex multimerons are presented in Figure S2. Similarly to the purely FM counterparts, in confined geometries (Figure S2b,c) a ”single” AFM Néel meronic state can be stabilized. This object is a trimeron resulting from three frustrated merons with overlapping cores, carrying in total a half-integer topological charge.

## Stability against External Magnetic Fields

The investigation of the response of topologically paired AFM Néel meronic pairs to magnetic fields is important to inspect stability aspects and to fingerprint subsequent potential nontrivial topological transitions.

The frustrated meronic textures survive to extremely high in-plane magnetic fields (>200 T). The case of an out-of-plane (OOP) magnetic field shows a rather rich impact on the explored spin textures. Therefore, here, we scrutinize in detail the latter scenario by focusing on three different AFM Néel meronic states (see Figure 2f–h).

As a prototypical chiral magnetic object, we consider the hexameron emerging either in the AFM Néel (Figure 2f) or in the spiraling AFM Néel states (Figure 2h) as well as the dodecameron (Figure 2g). For interfaces hosting the Fe layer, MnPdFe/Ir(111) and MnPd_2_Fe/Ir(111), we examined both cases: switching-off (solid bars in Figure 2e) and switching-on (dashed bars in Figure 2e) Mn–Fe magnetic interactions. A snapshot for the Mn–hexameron interfaced with ferromagnetic Fe spirals and skyrmkion is illustrated in Figure 2i.

While we were expecting the robustness of the unveiled meronic textures against external magnetic fields, we were intrigued by the annihilation of some hexamerons emerging in an AFM Néel background with experimentally accessible OOP fields, e.g., 10 T, in contrast to dodecamerons and hexamerons arising in a Néel spiraling state (red and green bars in Figure 2e).

**Figure 3:**
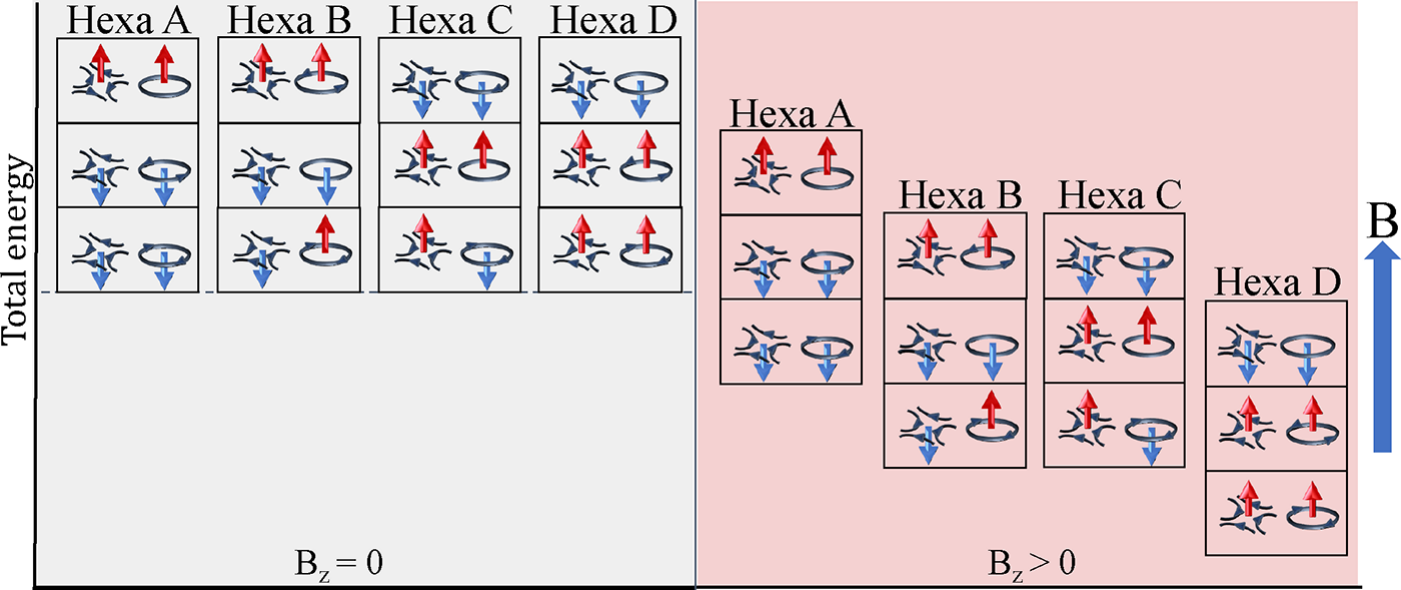
Topologically dependent response to the external magnetic field. Lifting the quadrupole degeneracy of the hexameron (Hexa A–D) upon application of an out-of-plane magnetic field. Each hexmeron is decomposed into the three sublattices with the illustration of the vortex nature of the meronic core constituents together with the core spin direction. Hexa D is the frustrated hexameron satisfying the ideal stability criterion against the magnetic field.

To obtain insight into the origin of the sensitivity of these magnetic states, hexamerons forming in an AFM Néel background, we scrutinize the sublattices’ topological distribution along with the spin orientation at each sublattice of the different hexameronic states shown in Figure 3 (see Figure S3 illustrating snapshots of the different hexamerons). As introduced earlier, there is a quadruple degeneracy for each hexameron in the absence of a magnetic field. The four states, denoted Hexa A–D and illustrated in Figure 3, can be distinguished by the vortex nature of their core constituents and the orientation of the core spins (see Figure 1k). A finite OOP field partially lifts the degeneracy and favors the hexameron, here Hexa D, with most spins pointing along the field direction (see also Figure S3). Among the four hexamerons, Hexa D will be the most robust to the applied field and therefore survives gigantic fields. The remaining hexamerons experience at some point magnetization switching to reach the optimal sublattice topological distribution defined by Hexa D. This requires a flip of the spins for at least one meron (antimeron) implying going through a topological charge transition, being a nontrivial process, during which the AFM meronic structure might encounter an unstable spin distribution, leading to the annihilation of the AFM meronic structure where the AFM vortex and antivortex start rolling toward each other and then collapse at a rather low magnetic field. If the transition occurs, however, the new magnetic state would be capable of surviving large magnetic fields similar to Hexa D.

However, the presence of Néel spirals in the background or additional pairs of AFM meronic textures (leading, for example, to dodecamerons) prevents the formation of unstable states within the topological transition induced by the magnetic field, which would lead to the collapse of the frustrated soliton. Effectively, a barrier is provided by enabling the rearrangement of the spins to acquire the desired topological state, which can withstand immense magnetic fields.

## Emergence Mechanism

We have identified that the formation of our frustrated AFM Néel meronic spin textures requires a strong AFM exchange coupling among the first nearest-neighbor atoms *J*_1_ (see Figure S1a–d). This coupling is responsible for the AFM Néel order of the spins, and it is through magnetic frustration that these solitons may arise. Additionally, another magnetic interaction is required to align the spins in the in-plane direction. This interaction can be provided by the in-plane MAE, *K* < 0, as observed in Mn/Ir(111), while for the other three magnetic systems studied, *K* prefers an out-of-plane orientation of spins (Figure S1e). However, the *z* component of the DMI vector (*D*_*z*_) plays a crucial role in aligning the spins in-plane, ultimately leading to the emergence of the AFM Néel meronic textures. In conclusion, to obtain our AFM solitons on a triangular lattice, an AFM *J*_1_ is required, along with either a finite *D*_*z*_ or an in-plane *K*.

**Figure 4:**
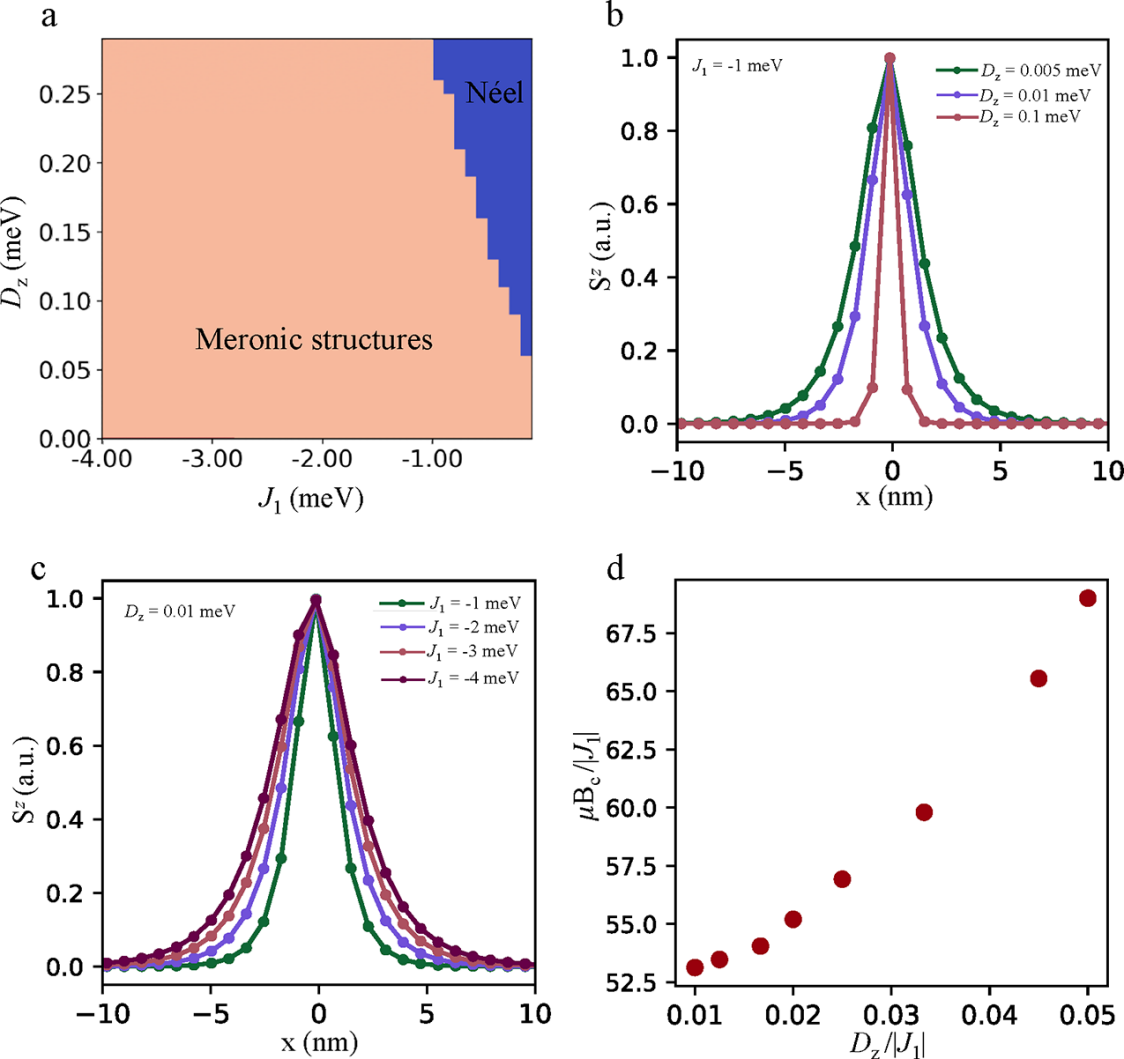
Minimal spin model for frustrated AFM Néel multimerons. (a) Phase diagram showing immediate emergence of hexamerons requiring an AFM nearest-neighboring magnetic interaction, *J*_1_, and an out-of-plane DMI component, *D*_*z*_. (b, c) Mutual impact of magnetic exchange interactions and DMI on the *S*^*z*^ profile of one of the vortices. (d) The associated critical field required for the annihilation of the multimeron as a function of the DMI.

To explore the fundamental mechanisms defining the stability of the spin textures, we built a minimal spin model that contains only AFM *J*_1_ and *D*_*z*_ because the latter played the main role in stabilizing the meronic textures in the four investigated Mn-based interfaces. The resulting phase diagram is shown in Figure 4a. While the ground state would have been a pure Néel state without *D*_*z*_, the latter enables the quick formation of frustrated merons. Increasing *D*_*z*_ enforces a stronger in-plane alignment of the spins, which reduces the size of the meronic constituents (Figure 4b and Figure S4). Clearly, the size of merons is dictated by a competition of magnetic exchange and DMI. Keeping *D*_*z*_ fixed while increasing the AFM *J*_1_ counteracts the effect of DMI and enlarges the meron core (Figure 4c).

Figure 4d presents the critical OOP magnetic field upon which the meronic texture, here Hexa D similar to that shown in Figure 3, is annihilated as a function of the OOP DMI component all normalized by the nearest-neighboring AFM exchange interaction. The obtained curve follows a quadratic dependence, highlighting that the DMI enhances the stability of the frustrated merons. In fact, the application of an OOP magnetic field counteracts the influence of the OOP DMI component by tilting the spins to the OOP direction, causing disruption to the in-plane alignment of the spins, imposed by the OOP DMI component, throughout the surrounding area, including the region spanning between the extremities of the hexameron, ultimately leading to its collapse. Consequently, the larger the OOP DMI component (smaller meronic cores), the larger the critical field required to destroy the AFM spin-swirling textures.

## Discussion

Our ab initio simulations uncovered nonsynthetic Néel-frustrated AFM meronic textures emerging in a realistic set of materials and interfaces. The newly unveiled nanoscale magnetic objects are hosted by a triangular Mn layer interfaced with an Ir(111) surface alone, or covered with a Pd overlayer, or separated from Ir by either a PdFe bilayer or a Pd_2_Fe trilayer, which all represent substrates that can readily be grown experimentally. The frustrated AFM states form hexamerons, composed of three FM meronic pairs each located at one of the three FM sublattices building up the AFM Néel background. Other solitons can emerge such as dodecamerons (12 merons) while confined geometries enable the stabilization of a frustrated trimeron.

We have observed that these AFM Néel meronic solitons survive high values of magnetic fields if the majority of the spins align in the direction of the OOP magnetic field. Otherwise, a transition of the sublattice topological charge occurs, leading to potential annihilation of the AFM solitons at experimentally accessible values of magnetic fields. To gain a better understanding of the characteristics of these AFM solitons, we provided a spin model that outlines the minimum set of magnetic interactions necessary to generate the detected AFM solitons.

The exploration and identification of AFM spin textures call for a multifaceted approach, encompassing both theoretical and experimental techniques. Recent theoretical advancements propose innovative methods like all-electrical detection based on tunneling spin-mixing magnetoresistance (TXMR).^59,71^ These theories also consider various operational modes^66^ and the potential enhancement through the introduction of atomic defects.^63^ Moreover, spin-polarized scanning tunneling microscopy offers the capability to resolve antiferromagnetic states with atomic precision.^72,73^ Similarly, remarkable progress has been achieved in X-ray magnetic microscopy^12^ and all-optical relaxometry, enabled by a scanning quantum sensor utilizing a single nitrogen-vacancy (NV) defect in diamond. These experimental methods have been successfully applied to study various synthetic AFM textures.^11^

We foresee the implementation of those frustrated AFM meronic spin textures in future spintronic devices, where a major aspect to be addressed is the ability to manipulate them because in general AFM spin textures are robust to magnetic fields. It is worth noting that AFM meronic structures, similar to the AFM topological solitons, despite their inherent immunity to magnetic fields, remain amenable to manipulation through external stimuli such as spin currents.^4,8,10,14,22,33,69^ While the manipulation of synthetic AFM spin textures using spin currents has been experimentally realized,^10,70^ theoretical predictions also extend to intrinsic AFM spin textures.^14,22,32^ In addition to spin-current-based manipulation, our study has demonstrated the sensitivity of certain hexameronic states to experimentally attainable magnetic fields. This suggests that the latter can be utilized as an external stimulus to control these frustrated AFM multimeronic spin textures, contingent upon the distribution of sequential topological charges across the sublattices.

Identifying new AFM solitons with a realistic existence scenario is at the heart of AFM topological magnetism. Our predictions can initiate the experimental discovery of intriguing intrinsic frustrated multimeronic textures, which can be delineated in various topological sequences. It remains to be explored how such spin states can be implemented and designed in AFM spintronic devices. Certainly, the thin films being proposed provide a solid platform for AFM meronic textures with a potential impact on information technology.

## Methods

In this study, we conducted a systematic investigation to explore the magnetic structures that can be hosted by the magnetic layers of our four layered systems. Our approach involved a 3-fold procedure, combining ab initio calculations with spin atomistic dynamics. The details of this procedure are outlined below.

To simulate the magnetic properties of our magnetic layers, we utilized in a first step the Quantum-Espresso computational package.^74^ The calculations employed projector augmented wave pseudopotentials sourced from the PS Library,^75^ and the self-consistent calculations were performed with a k-mesh of 28 × 28 × 1 points for the unit cell. The layers were arranged in an fcc-stacked configuration along the [111] direction (Figure 2a–d). The relaxation parameters were then extracted, revealing the relaxation percentages of the different layers in relation to the ideal interlayer distance in the Ir-based systems. Specifically, for Mn/Ir(111), the relaxation percentages were 2.3% and −3.4%; for PdMn/Ir(111), they were 8.6%, 10.3%, and −2.3%; for MnPdFe/Ir(111), the percentages were 4%, 5.2%, 8.1%, and −1%; and for MnPd_2_Fe/Ir(111), they were 5.9%, −4%, 8.2%, 8.2%, and −0.7%, for each layer, respectively. Here, positive (negative) values indicate atomic relaxations toward (away from) the Ir surface.

After establishing the geometries of the various magnetic systems, we conducted in a second step a detailed investigation of their magnetic properties and interactions using the all-electron full-potential relativistic Korringa–Kohn–Rostoker (KKR) Green function method, implemented in the JuKKR computational package,^76,77^ in the local spin density approximation. Each of the four magnetic systems consists of a slab with 30 layers. In the case of Mn/Ir(111), the slab consists of 5 vacuum layers + 1 Mn layer + 20 Ir layers + 4 vacuum layers. For PdMn/Ir(111), the slab comprises 4 vacuum layers + 1 Pd layer + 1 Mn layer + 20 Ir layers + 4 vacuum layers. In the case of MnPdFe/Ir(111), the slab includes 3 vacuum layers + 1 Mn layer + 1 Pd layer + 1 Fe layer + 20 Ir layers + 4 vacuum layers. Lastly, for MnPd_2_Fe/Ir(111), the slab is composed of 2 vacuum layers + 1 Mn layer + 2 Pd layers + 1 Fe layer + 20 Ir layers + 4 vacuum layers. To perform the calculations, the momentum expansion of the Green function was truncated at . Self-consistent calculations were conducted using a k-mesh of 30 × 30 × 1 points. The energy contour consisted of 23 complex energy points in the upper complex plane, and it incorporated 9 Matsubara poles. To extract the Heisenberg exchange interactions and Dzyaloshinskii–Moriya (DM) vectors,^78−81^ we employed the infinitesimal rotation method.^82,83^ For this extraction, we used a finer k-mesh of 200 × 200 × 1 points.

After extracting the magnetic parameters for our magnetic atoms from first-principles, we employ the Landau–Lifshitz–Gilbert equation (LLG) implemented in the Spirit code^67^ to explore the magnetic properties and complex states. This exploration involves minimizing the two-dimensional Heisenberg Hamiltonian on a triangular lattice. The Hamiltonian comprises several terms, including Heisenberg exchange coupling, Dzyaloshinskii–Moriya interaction (DMI), magnetic anisotropy energy, and the Zeeman term. The energy functional of the system can be described as follows:
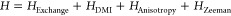
1with
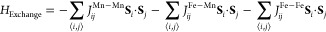

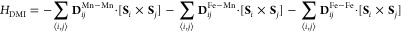

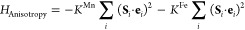

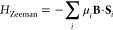
where we assign indices *i* and *j* to denote specific sites, each associated with a magnetic moment. The magnetic moment is represented by the unit vector **S**. The Heisenberg exchange coupling strength *J*_*ij*_^X–Y^ describes the interaction between an atom X on site *i* and an atom Y on site *j*, where a negative value indicates an AFM interaction. Similarly, we use notation **D** for the Dzyaloshinskii–Moriya interaction vector, *K* for the magnetic anisotropy energy, and μ_*i*_**B** to represent the Zeeman coupling to atomic spin moment μ at site *i*. It is important to note that the Fe–Mn and Fe–Fe interactions are considered only in the MnPdFe/Ir(111) and MnPd_2_Fe/Ir(111) systems. For our spin atomistic simulations, we adopt both periodic and finite boundary conditions to model the extended and confined two-dimensional system, respectively, with cells containing 249^2^, 300^2^, and 390^2^ sites.

## Data Availability

The data needed to evaluate the conclusions in the paper are present in the main manuscript and the Supporting Information. We used the following codes: Quantum ESPRESSO which can be found at https:/www.quantum-espresso.org/download, SPIRIT can be found at https://github.com/spirit-code/spirit, and the KKR code is a rather complex ab initio DFT-based code, which is in general impossible to use without proper training on the theory behind it and on the practical utilization of the code. We are happy to provide the latter code upon request.

## Supporting Information Available

Magnetic interactions among Mn atoms for the investigated systems; different AFM multimeronic textures that emerge in the Mn layer; illustration of the initially degenerate topologically different hexameronic states; and impact of the nearest-neighboring magnetic interaction on the size of the AFM vortex–antivortex pairs (PDF)
