# Supplementary material for: Intrinsic Néel Antiferromagnetic Multimeronic Spin Textures in Ultrathin Films
Source: J Phys Chem Lett. 2023 Sep 29;14(40):8970–8. doi: 10.1021/acs.jpclett.3c02419 (PMC10577774; doi:10.1021/acs.jpclett.3c02419)
Supplement: Supplementary file 1 — jz3c02419_si_001.pdf [file jz3c02419_si_001.pdf]

Supporting Information

Intrinsic Néel Antiferromagnetic Multimeronic Spin-Textures in Ultrathin Films

Amal Aldarawsheh *[1,2],[†] Moritz Sallermann[1,3,4],[†] Muayad Abusaa[5],[†] and Samir Lounis*[1,2*,†]

†[1] *Peter Grünberg Institute and Institute for Advanced Simulation, Forschungszentrum Jülich and JARA, D-52425 Jülich, Germany*

‡[2] *Faculty of Physics, University of Duisburg-Essen and CENIDE, 47053 Duisburg, Germany*

¶[3] *RWTH Aachen University, 52056 Aachen, Germany*

§[4] *Science Institute and Faculty of Physical Sciences, University of Iceland, VR-III, 107 Reykjavík, Iceland*

‖[5] *Department of Physics, Arab American University, 240 Jenin, Palestine*

E-mail: a.aldarawsheh@fz-juelich.de,m.sallermann@fz-juelich.de,muayad.abusaa@aauj.edu,s.lounis@fz-juelich.de

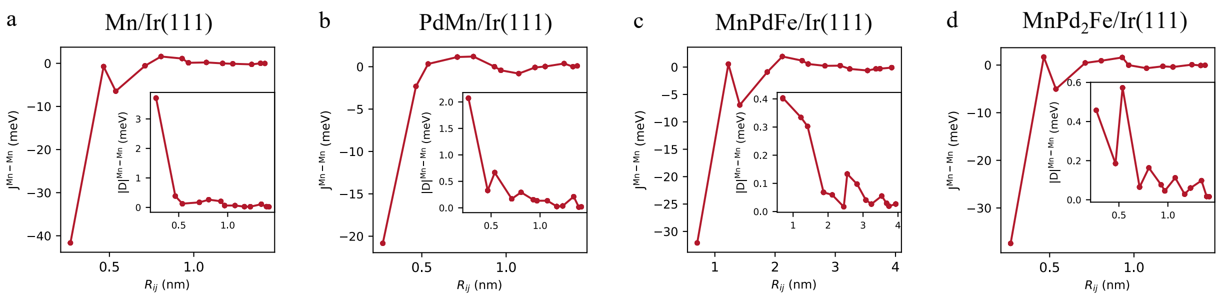

e

	MnIr	PdMnIr	MnPdFeIr	$MnPd_2FeIr$
Mn μ	3.4	2	3.7	3.8
Mn K	-0.21	0.76	0.5	0.18
Magnetic moment (μ) and Anisotropy strength are in meV				

Figure S1: Magnetic interactions among Mn atoms for the investigated systems: The Exchange interactions values as a function of distance for MnIr/(111) **a**, PdMn/Ir(111) **b**, MnPdFe/Ir(111) **c** and $MnPd_2Fe$/Ir(111) **d**, insets show the DM interaction values as a function of distance. **e** The spin moment per Mn atom (μ) in μ_B and the magnetic anisotropy energy (K) for Mn layer for the inspected systems.

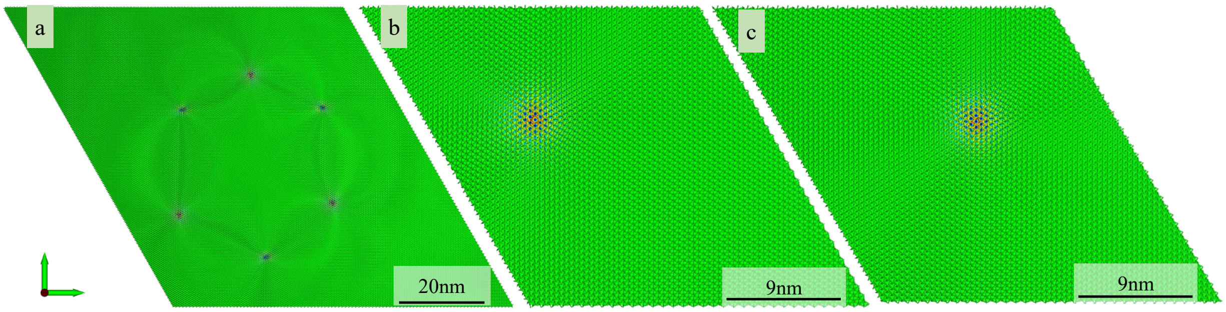

Figure S2: Different AFM multimeronic textures that emerge in the Mn layer: **a** Excited state with six AFM meronic structures. **b,c** Single AFM meron, antimeron form on confined geometries.

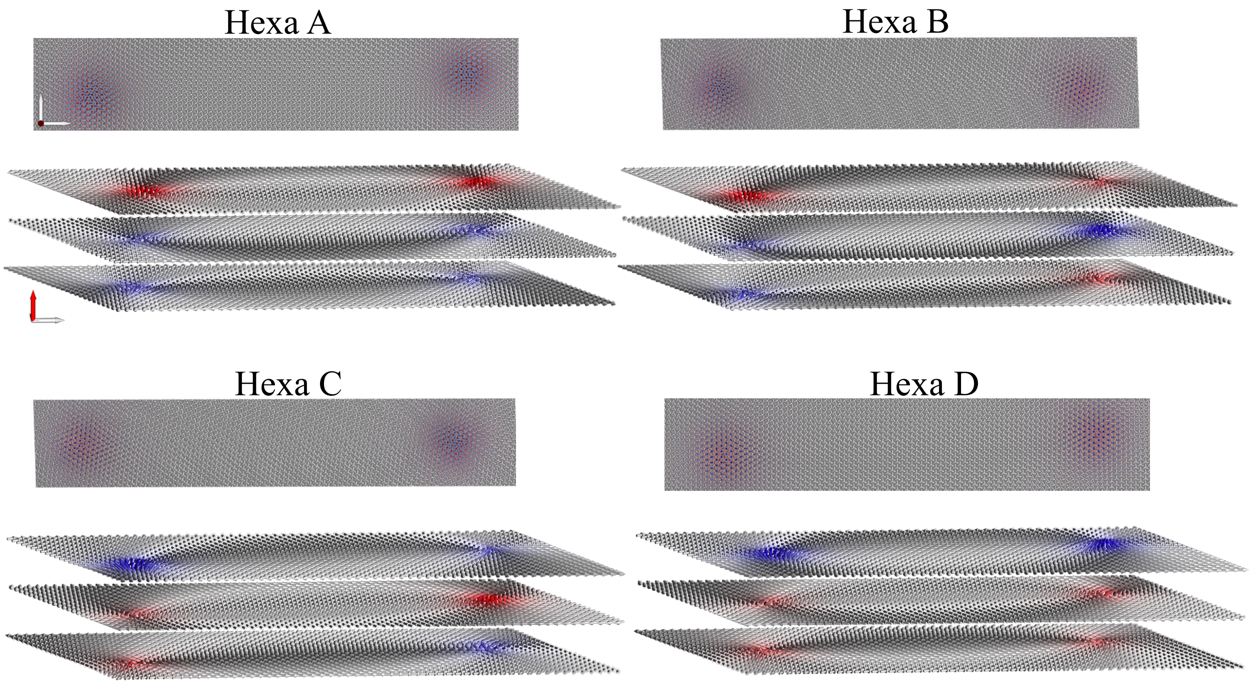

Figure S3: Illustration of the initially degenerate topologically different hexameronic states: Snapshots showing the hexamerons (upper) and their FM meronic decomposition at the three sublattices (lower).

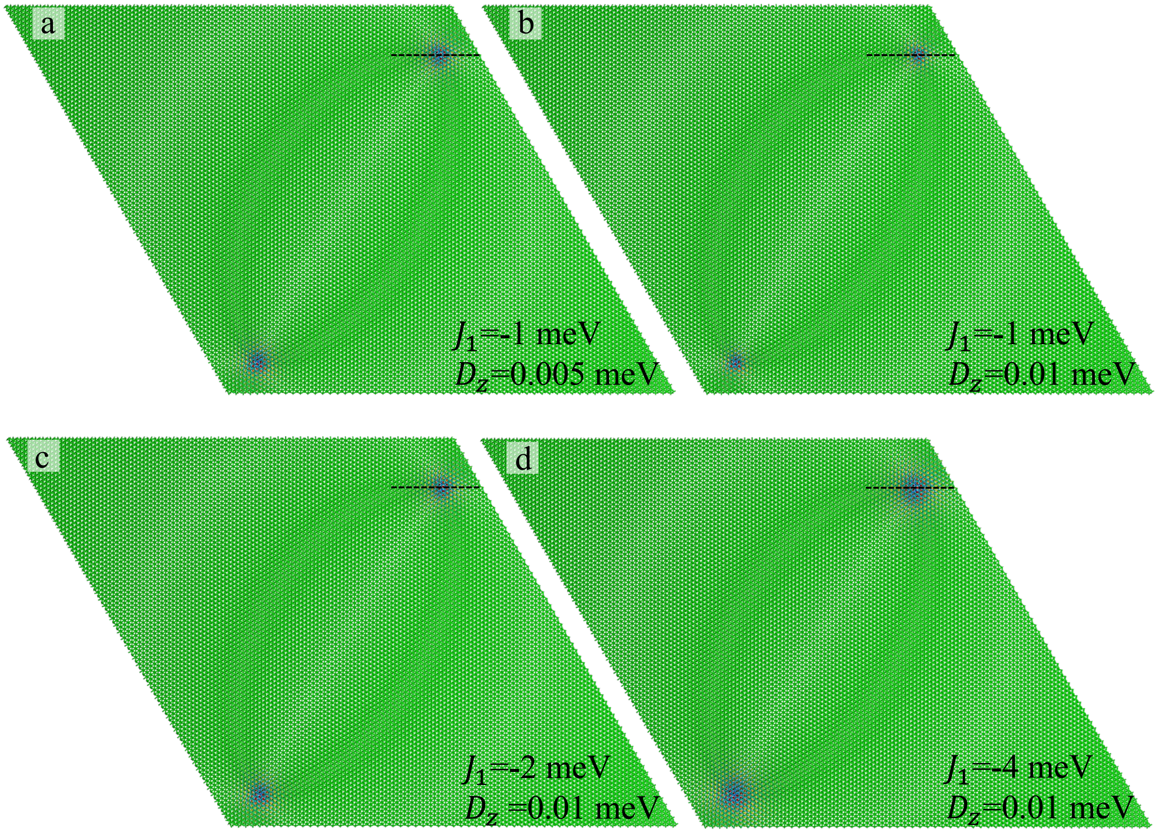

Figure S4: Impact of the nearest neighboring magnetic interaction on the size of the AFM vortex-antivortex pairs: Results obtained with a minimal spin model with the magnetic exchange interaction J_1 and out-of-plane component of DMI D_z. snapshots for **a** D_z=0.005 meV, J_1=-1 meV, **b** D_z=0.01 meV, J_1=-1 meV, **c** D_z=0.01 meV, J_1=-2 meV, and **d** D_z=0.01 meV, J_1=-4 meV.
